# Maternal, newborn and child health needs, opportunities and preferred futures in Arusha and Ngorongoro: hearing women’s voices

**DOI:** 10.1186/s13104-015-1776-6

**Published:** 2015-12-12

**Authors:** Pammla Petrucka, Sandra Bassendowski, Marie Dietrich-Leurer, Cara Spence-Gress, Zenath Athuman, Joram Buza

**Affiliations:** University of Saskatchewan, 4400 4th Avenue #100, Regina, SK S4T 0H8 Canada; Mama Kwanza Socio-economic Health Initiative, Arusha, Tanzania; Nelson Mandela African Institute of Science and Technology, Arusha, Tanzania

**Keywords:** Tanzania, MNC health, Community dialogues, Arusha, Ngorongoro Conservation Authority, Baseline assessment

## Abstract

**Background:**

With the approaching sunset on the Millennium Development Goals (MDGs), Tanzania continues with its final national push towards achievement of MDG #4 and MDG #5. The Mama Kwanza Socio-economic Health Initiative (MKSHI) was introduced in the hope of contributing to improving maternal, newborn, and child health in Arusha and Ngorongoro. The MKSHI project is a holistic, inter-sectoral approach to maternal, newborn, and child health which aligns with the Government of Tanzania’s *Vision 2025*. At the project onset, a baseline assessment was conducted to launch ongoing benchmarking, monitoring, and evaluation of the project’s impacts and implications. The aim of this baseline assessment was twofold. First it was to determine the state of maternal, newborn, and child health in the two project sites. Second it was to ensure that a baseline of key indicators was established as well as identification of unique indicators relevant to the populations of interest.

**Results:**

The baseline study was a mixed methods approach to identify maternal, newborn, and child risk factors and indicators in the two target sites. This paper focuses on the qualitative methods and findings. The qualitative component included a series of five community dialogue meetings and thirty-seven individual/dyad interviews with women, providers, and stakeholders. Initially, community meetings were held as open dialogues on maternal, newborn, and child health issues, opportunities, and preferred futures. Individual/dyad interviews were held with women, providers, and stakeholders who held unique information or experiences. Both community dialogue and interview data was analysed for themes and guiding or critical comments. Three over-arching findings emerged: *What took you so long to come? How do we know what you know?* and *How will it change for our daughters?*

**Conclusions:**

Participant voices are vital in ensuring the achievement of local and global efforts and preferred futures for maternal, newborn, and child health services. This study contributes to the inclusion of women in all aspects of the planning, implementation, and delivery of maternal, newborn, and child health services in the target areas and beyond.

## Background

Globally, women and children continue to die of preventable and/or treatable causes which require simple, proven, low-cost interventions [1]. The following list reflects a number of these issues: one in ten hunger related deaths are due to disasters (i.e. drought, floods) with the remainder due to systemic, chronic, or recurring situations; one in eight people globally are suffering from chronic under-nutrition with 98 % of these located in developing countries; one in six babies are low birth weight due to prenatal malnutrition; one in four people in Africa is hungry; one in three people in developing countries are deficient in vitamins and minerals; and one in two child deaths are related to nutritional status (~5 million annually) [2–4].

Diseases, such as pneumonia, diarrhea, and malaria, account for over 40 % of deaths in children under 5 years of age [5]. Globally, diarrhea is the second most common killer in this age group [6]. Third on the list of causes is malnutrition/undernutrition, including micronutrient deficiencies, which contribute to an estimated 30 % of deaths, despite optimal feeding guidelines, such as the Global Strategy for Infant and Young Child Feeding [7]. In sub-Saharan Africa, the number of underweight children is expected to exceed 43 million in 2015, with significantly more females experiencing Vitamin A deficiency and stunting, which impact on health, educational, and economic futures [8]. Exclusive breastfeeding alone within the first 6 months of life would save about 1/3 of a million children each year worldwide, yet only 30 % of infants in sub-Saharan Africa are exclusively breastfed [9].

Within the struggle to achieve maternal, newborn and child health (MNCH) the Mama Kwanza Socio-economic Health Initiative (MKSHI) was conceived. The MKSHI project, which grew from a long term relationship between Tanzanian and Canadian partners, expands services across sectors for MNCH in select communities in Tanzania. The project aims at demonstrating and informing future models for innovative, quality, and sustainable programs for most vulnerable populations in the Arusha and Ngorongoro Districts. The MKSHI focuses on a social determinants approach dedicated to addressing Millennium Development Goals (MDGs) #4 and #5 for most vulnerable women and children in Arusha (an urban/peri-urban catchment) and Ngorongoro (remote) Districts, Tanzania. In Tanzania, these two specific maternal newborn and child health (MNCH) goals have remained a challenge. As of 2013, Tanzania’s infant mortality rates (IMR), neonatal mortality (NM) and maternal mortality rates (MMR) remain high at 38 per live birth, 21 per 1000 live births (i.e., over 48,000 per year/17,000 as first day deaths), and 460 per 100,000 (i.e., approximately 8500 deaths) respectively [10]. Three critical elements considered within the MKSHI are skilled birth attendance, family planning, and post-natal care.

Currently in Tanzania, about half of women are unable to access skilled birth attendance (49 %); family planning (48 %); and post natal care (31 %) [11]. Countdown to 2015 [10] noted that these inequities are embedded in poverty issues with the poorest quintile having attended births 31 % of the time versus 90 % attendance for the wealthiest quintile. As the Government of Tanzania’s Vision 2025 [12], the health sector strategic plan III [13], and other health, social services, and strategic national agendas have articulated these realities, the MKSHI project has embedded these into its planning and programming.

This paper reports on the method, results, and learnings from the baseline assessment in Arusha and Ngorongoro, Tanzania. Primarily the intent was to determine the state of maternal, newborn, and child health in the two project sites. Secondly it was to ensure that a baseline of key indicators was established as well as identification of unique indicators relevant to the populations of interest to be tracked and impacted upon during the MKSHI.

## Methods

Our selection of a method was based on the goal of being able to ‘validate’ that the women/communities with whom we were working either mirrored or differed from those represented in government documents—specifically The *Tanzanian Atlas of Maternal Health, Child Health and Nutrition* [14]. We also sought baseline information on select indicators to enable ongoing assessment of the MKSHI impacts on the women/communities over the course of the project.

Ethics approval was obtained from the University of Saskatchewan Research Ethics Board (BEH 11-328) and affirmed through the Nelson Mandela Institute of Science and Technology. Each tool was pilot tested or expert reviewed. The community dialogue guide, and client interview were piloted at a partnering site as it was felt that this site had a highly diverse population (i.e., illiteracy, poverty, and ethnicities). The health provider and stakeholder interview guides were expert reviewed by members of the project team.

Adjustments to the draft tools were made based on these feedback experiences resulting in a Community Dialogue guide and three separate interview guides (for clients, health providers, and stakeholders).

### Sampling

The qualitative sampling was situational and maximized representation as well as identified unique informants and extreme cases.

For the community dialogue meetings, women were recruited by open invitation (using word of mouth and posted messages) in a convenience sampling approach. Women were asked to notify the local clinic at least 2 days prior to the intended session in order to allow planning for seating and refreshments. The clinics were asked to limit the number of women to 25 participants with assurance that if that number was exceeded we would offer subsequent dialogue meetings. It was agreed that we would not hold more than two sessions per site due to time limits on completion, and likelihood of saturation. The participation by community is shown in Table 1. In no situations was a second session held; however, in two communities in Ngorongoro, more than 25 women attended the sessions and the decision was made on the ground to proceed. A group consent process was facilitated by the local leader at all sessions.

**Table 1:** Sampling for community dialogue meetings

Site	Number of attendees
Philips	17
Mrombo	14
Olbalbal	47
Misygio	21
Alilayli	33
Total	132

For the client interviews sampling was based on identification of individuals from each community dialogue meeting (by the research team) who had unique or specific contributions that required further exploration. From there, a snowballing process linked us to other individuals in their communities (who may or may not have participated in the community dialogue session) (see Table 2). All interviewees were provided with a consent form in Swahili and given a verbal explanation by a local staff (i.e., interpreter) employed by the MKSHI clinic. Voluntary consent was given either in writing or verbally by the interviewee.

**Table 2:** Sampling for individual interviews for women

Site	Number of interviewees
Philips	3 (2 Participants; 1 non participant)
Mrombo	1 (1 Participant)
Olbalbal	3 (1 Participants; 2 non participants)
Misygio	1 (1 Participants)
Alilayli	2 (1 Participants; 1 non participants)
Total	10

### Data collection and entry

Data collection and entry was guided by the following principles of:

- Using local language (which meant local data collectors).
- Ensuring real-time or near real-time capture and entry (to minimize loss of ideas or insights).
- Considering respectful and relevant data capture procedures (to avoid unnecessary time and information).

Three steps were followed in this baseline assessment.

- Step one: training of community researchers

Three community health workers were trained—one for the Arusha District and two for the Ngorongoro District. The workers received a 12 h training session on community based research and the specific tools for this project. These individuals participated in the pilot testing exercise at Mrombo in order to both experience the process and to contribute to the revision of the tools. They were not involved in the health provider or stakeholder interviews.

- Step two: fieldwork

The fieldwork included logistical preparations as well as data collection and entry efforts. The entire group participated in the first site (Philips) in order to model consistency and give the opportunity for any questions or methodological adjustments. The team was then split with one group continuing in Arusha and the other beginning their fieldwork in Ngorongoro.

Data entry was done by the onsite research group. Teams would conduct data collection on Mondays, Wednesdays, and, if necessary, Fridays, with data entry occurring on alternate days.

- Step three: validation/quality monitoring

Although listed independently and sequentially, there were constant efforts to compare and contrast the findings across sites and tools. The quality monitoring of the data was important to ensure all data elements were being captured, and correctly reflected. The team attempted to have weekly meetings to share their experiences, but the amount of fieldwork overwhelmed them and only one meeting occurred at week 2. Hence, final data integrity reviews was left with the lead research team.

## Results and discussion

### Overview

Through community dialogues and interviews with select women, the research team heard a range of personal stories, anecdotal recollections, and reflective thoughts on the state of maternal, newborn, and child health care, programs/services, and providers. The community dialogues included 132 women in five communities ranging from urban to peri-urban to remote locations. During these sessions, the focus was on perceptions, experiences, and preferred futures of MNCH. In addition, ten women were recruited to provide brief interviews. Six individuals identified from the community dialogue groups were selected as:

- Two individuals requested an interview as they had a story/experience they did not wish to share in the group.
- Three individuals shared a story or issue that was flagged by the team as unique and meriting further exploration.
- One individual was a non-health professional who had insights that the team felt could contribute to an added dimension.

Following individual interviews, the participants were asked if they wished to recommend someone else from their community who might have additional insights or perspectives on MNCH. Although six recommendations were received only four agreed to participate. Because these women constituted a small number of participants, and due to the nature of their stories, we choose not to locate or name them in order to protect their identities.

Although we intended to use the same instrument as the community dialogues, the original core group of women wanted to focus on experiences and it was decided that this should be the focus of the individual interviews for all cases.

#### Perceptions of MNCH

The women were asked about their perceptions or thoughts about quality of MNCH services, and providers. In inviting them to this discussion, they were told that we were most interested in their personal and community viewpoints. The participants are recognized by abbreviations of their names and which district they are associated with. Their responses, across all sites, fell into two major categories—maternal and child issues and gender influences. Within each of these categories a number of themes and/or sub-themes were identified and will be discussed herein.

##### Perceptions of maternal and child issues—what we heard

The women stated that MNCH was perhaps the most difficult aspect of their lives. Whether the challenges were about risks, finances, or physical aspects, the women spoke clearly about what type of services they might have to help them “live through this time” (CC Arusha).

it (pregnancy) should be a happy time, but for us there is fear and worry about how things will go or not. Maybe we have had a sister die or lost a child before, so we know the risks. So how can this be happy? (N. Arusha).

women have lived and died bringing children to this world…but when it is your turn you cannot help but be a bit afraid how you will make out. (L. Ngorongoro).

Women in the Arusha District spoke about the perception that the woman has to take charge and know as much as she can about MNCH. They spoke of knowing the difference between a fever and malaria or when it is time to take their child for care. There was a sense of pride and also a sense of necessity in their discussion of this topic. They felt that “if we not know who else would care like a mother?” (B. Arusha). The emphasis was on the imperative of a mother being aware of the health care needs and services in order to access these in a timely manner if needed.

Many women perceived that there is no one to help them. Some spoke of their absent husbands who provide minimal support or presence throughout the pregnancy, delivery, and child-rearing. The attitude of “they are just men” (D. Ngorongoro) was consistent across sites. Further, the lack of health providers and health facilities was cited as further evidence that “we are more alone” (M. Ngorongoro).

Through thematic analysis, three themes were extracted in relation to perceptions of MNCH—*it’s about survival; knowing as a mother should; and we are on our own.*

##### Perceptions of gender influences—what we heard

The women told us that MNCH is a women’s issue and that very few men would see it any other way. Most of the women spoke of not expecting to have their husbands by their side during labour and delivery. Women even spoke about being “left at the clinic when the time comes” (B. Arusha).

He will not care about even coming to the ante-natal clinic, so I go without him. It is just the way it is and nothing can change that. So I walk the 3 km with others from the village who are too pregnant. (L. Ngorongoro).

The issue of decision making and who decides on health care issues was discussed. At times there were extremes of emotion (both agreement and disagreement) on this issue. Most agreed that men held the ultimate or final decision in matters of money and most resources. This was seen as impacting on the type of care that might be possible. A general consensus was heard on the shifting of this power, especially in the Arusha District. The women spoke of “if only I had the shillings in my pocket” (A. Arusha) or “when it is my money” (R2 Ngorongoro), clearly indicating a gender difference in control of financial resources, which is, at times, vital for accessing MNCH.

I needed to go to the (private) hospital, but my husband said no go to the (government hospital) instead. They did not have the working ultrasound, so we came back home. I did not have the test and now my baby is very sick all the time. (A. Arusha).

My husband knows that I need money every month to check the baby for growing and weighing. He does not question me but gives it so that I can take him. I think he has learned that this is good for our child.” (P. Arusha).

With respect to perceptions of gender influences, two themes emerged—*women present/men absent* and *women do/men decide*; as well as one minor sub-theme emerged which was attached to the second major theme—*lack of resources equals lack of access*.

#### Experiences of MNCH

The women shared their experiences and stories regarding MNCH services and providers. The responses, across all sites from the community dialogues, fell into three major categories—experiences of care, roles/responsibilities, and family planning.

##### Experiences of care—what we heard

Of all the discussions, this topic had the most input and was highly animated. The women were passionate about their care needs, explicit about their care experiences, and concerned about care quality. With a seemingly singular voice they all indicated a need for more MNCH care—and, more importantly, quality, local MNCH care. The women told of distances traveled, lack of health providers, lack of supplies, and mistreatment by health providers (i.e., rudeness, yelling).

to go for care so many times before and after the baby comes… but sometimes they are so busy that we just wait and wait only to leave not seen. (R2 Ngorongoro).

At dispensaries and even health centers don’t have doctors and so few nurses…we go for care and find they don’t have staff or supplies. So I don’t know if it is safe? I went to get my baby immunized and was told the government had run out of the medicines and no one knew when we would get the new medicines. So now I worry that my baby will get sick. (P. Arusha).

Two major themes arose with respect to experiences of care—*not just more but better care* and *so little for so many*. There was one sub-theme related to the first major theme which was *do as you are told*.

These findings were mirrored in the individual interviews. Three of the women from the community dialogues and one from the snowballing capture focused on medical stories. In these instances, the women spoke about issues of malpractice, negligence, and systemic flaws. Three of the women’s stories related to care issues that spoke to professional misconduct and failure to provide for care. One of the women spoke of how she came for services because she was concerned that she felt her baby was not moving as much. The staff at the clinic criticized her for wasting their time and sent her back home nearly 17 km. Within 3 days she was experiencing a high fever and foul discharge. By the end of the ordeal she not only lost her unborn child, but would never again be able to conceive. Another told of watching her sister hemorrhage to death after delivery because there were 7 other active deliveries and only one midwife and two nurses. The lack of staff and, in her opinion, their exhausted state led to the death of an only sister, new mother, and young wife. The third woman talked about her experiences due to female circumcision. She had begged for a caesarian section, but instead experienced severe tearing which took months to heal and have left her unable to have comfortable sexual experiences. Her concern was for other women who have been traumatized in this way and a plea that someone work on reducing this risk. The final woman told the team about the differences in care received by women based not on need but on financial resources. As a social worker she struggles to see the poor and less educated treated at the lowest level facilities, often with the lowest educated staff. Yet, it is these women who require additional resources, supports, and treatments.

Two additional major themes were extracted respecting experience of medical care—*failing to provide care* and *failing to care*.

##### Experiences of roles and responsibilities—what we heard

Although there was some obvious overlap with the perceptions findings (especially related to gender), this category emerged strongly. The women shared their lived experiences of multiple roles, responsibilities, and expectations which makes taking care of their personal and children’s health very challenging. There were comments about how culture affects these roles and responsibilities and makes change seem impossible. Sometimes the cultural ideas about MNCH conflict with the health system. For example, the Maasai women described that half rations are provided to the pregnant woman with the intention of reducing the birth weight of the baby theoretically causing less difficulties in labor.

we are responsible for everything…the home, water, children, everything even the parents…and then we have to worry about the coming baby. It is too much. (S. Ngorongoro).

Two major themes were derived in relation to experiences of roles and responsibilities—*responsible for everything and everyone* and *MNCH as cultural*.

Two of the women from the community dialogues and two from the snowballing capture participating in the interviews focused on social stories. The women spoke about issues of family violence, poverty, and family interference. Their stories not only enriched the understanding of gender and cultural elements that had been previously shared, but focused on the outcomes of these elements.

Two women told of the near daily assaults and violence perpetuated on them throughout their pregnancy and their fears of dying. They told us that there were no resources for such family violence and the issues were very real for many women. It was almost implied that this was the norm in some groups. One woman spoke of how poverty had cost her three children. She told us how each child could have lived with medicine or proper food, but the children were not valued. Her story was filled with a plea for programs to help women like her have healthy babies. She spoke of feeling “less a woman” in the eyes of society because she could not have healthy children. The final woman in this group spoke about the family pressures of Tanzanians where it is expected to have many children and even many wives.

She said that many woman keep having babies at risk to their own lives and their babies’ lives just because it is expected. She said some husbands will even leave or marry another wife if they feel you are not productive enough.

Three additional major themes were extracted respecting experiences of roles and responsibilities—*it’s a violent time; further marginalized;* and *when family values don’t value.*

##### Experiences of family planning—what we heard

A number of women talked about family planning and choices. This topic included many side conversations and even debate more than dialogue. Some were very open that they used family planning methods; others spoke of “(getting) the needle without him (my husband) knowing” (O. Arusha). For those in favor of family planning, they spoke about how they believed their lives and their children’s lives would be better. There were discussions about the cultural (i.e., “children bring wealth to my husband”, S. Ngorongoro) and religious (K. Arusha) aspects of family planning. There was limited talk about using birth control to have more time (birth spacing) between pregnancies, but most women talked about no more children. All of the women stated that their husbands would not take the lead in family planning so it was up to them to decide.

After my last child the midwife asked me about family planning. She went to my church, so I asked her but God does not want this. She tells me God wants healthy children and their mommies. I think she is rights, so I take the needles now. (R. Arusha).

In terms of experiences of family planning, two major themes were extracted—*making the cover overt (i.e., revealing family planning)* and *family planning as a means to family progress.*

A final aspect of the experience of family planning related to ethical issues in care. In this instance, one woman from each source contributed to the ethical stories. The women spoke about abortion and post-abortion care. We recognize that abortion is not legal in Tanzania, however, that does not mean such procedures are not being conducted often in the most rudimentary of conditions. The women both told of personal or known cases of sought abortions. In both stories, they spoke of how the MNCH system must recognize and address this gap. Women and young girls, according to them, are being forced to risk their lives. They stated that the health care system does not treat women who have had an abortion very well. Both women questioned how ethically the health system could not respond to this need.

One additional major theme was extracted—*desperately seeking ethical MNCH care*.

#### Preferred futures for MNCH

The women generally took time to think about what they wanted to see in their future MNCH palette. There were a number of women who described system improvements and this launched a number of discussions about what needed improving, what should come first, and where this should happen.

##### Preferred futures—what we heard

There was agreement on the need for more health providers (especially nurses and midwives), more equipment and supplies (such as gloves), and more facilities. Some women, especially from Ngorongoro, felt there was a need for transportation both to get to appointments and for health emergencies. Where the conversation became very interesting and touching was in one site in Arusha District where the women focused on making a difference in MNCH for the future women. It was a passionate plea to make changes now so that women and babies would not die.

I don’t think I am any better off for (MNCH) care than my mother was…really I think we stayed the same or maybe even are worse. I don’t know if this can be fixed but I want to see it better for my children and grandchildren. Women need to work together for that. (S. Arusha).

Two major themes were extracted from this aspect of the community dialogues—*envisioning a resource filled future* and *for our daughters*.

### Reflection on findings

#### Thematic summary

A brief review of the themes is highlighted in Table 3. Although frequency counts are reported these should not be interpreted as priorities or levels of importance. Rather, the intent was to show an overall distribution of responses across topic and themes.

**Table 3:** Qualitative theme frequencies

Topic	Themes/subthemes	Frequency	Total
Perceptions	It’s about survival	14	57 (28.4 %)
	Knowing as a mother should	10	
	We are on our own	9	
	Women present/men absent	10	
	Women do/men decide	8	
	Lack of resources = lack of access	3	
Experiences	Not just more but better care	38	97 (48.2 %)
	Do as you are told	9	
	So little for so many	15	
	MNCH as cultural	13	
	Responsible for everything and everyone	6	
	Making the covert overt/revealing family planning	12	
	Family planning as family progress	4	
Preferred futures	Envisioning a resource filled future	7	11 (5.5 %)
	For our daughters	4	
Socially speaking	It’s a violent time	6	12 (6 %)
	Further marginalized	4	
	When family values don’t value	2	
Medically speaking	Failing to provide care	12	17 (8.4 %)
	Failing to care	5	
Ethically speaking	Desperately seeking ethical MNCH care	7	7 (3.5 %)
			201

#### Situating the findings

Many study findings align with the existing literature related to MNCH globally and in sub-Saharan Africa.

More than 1000 women die each day during pregnancy and child birth mainly due to poor access to effective interventions [15] which resonates with the perceptions and fears expressed by the participants in this study. In terms of the MDG #4 and #5, it was important to recognize that these participants continue to be ‘left behind’ in terms of both child and maternal health care and status.

The participants in this study indicated that access to health care and appropriately trained providers is imperative and more notably in their absence the consequences are grave. This is reflected in the work of the African Union [16], which found that African women and children continue to die from three key delays: seeking care, reaching the health facility, and receiving appropriate care by health providers. Again, these are issues and contributing factors which have contributed to the failure of Tanzania’s health system to fully realize the MDG #4 and #5 goals.

In the present study, the role of gender as impacting MNCH outcomes, which is reflected in the literature respecting how social and cultural beliefs are fundamental to the engagement of men in the health of women and children [17]. Globally, gender inequality is one of the most important drivers of poor health and wellbeing outcomes for women and their children [18]. The emphasis is on the need for MNCH interventions aimed at active roles of men in improving and supporting MNCH [19, 20].

Although many authors [21, 22] speak of the primary and essential role of nurse and midwives in the care of laboring women. However, the literature is replete with description of poorly remunerated staff [23, 24], lack of equipment and supplies [25], and high workloads [23, 26]. These conditions amongst others have contributed to an alienated and demoralized workforce—no longer able to care and often leading to abuse in the patient-nurse relationship ranging from physical to emotional to verbal attacks [27, 28]. Mbabazi [29] reports a number of negative labour experiences which sound ominously similar to those reported by participants in the current study.

A number of study participants spoke of the need for safe abortion care in Tanzania. Globally, there are over 75 million unintended pregnancies which contribute to about 22 million unsafe abortions [26]. The challenge lies in the unmet need for contraceptives, most often in developing countries [26]. Although nearly one-third of maternal deaths are related to unsafe abortion, only 5 % of health facilities in Tanzania provide post abortion care [15].

As the participants envisioned the future of MNCH as a well resource health system which is highly responsive. According to Mwaikambo [30], there is a need to address two critical aspect of MNCH—health system factors and non-health system factors. Within health system factors, the emphasis lies with improved health infrastructure, increased skilled human resources, access to modern family planning, and appropriate equipment and supplies, plus a strengthening of policies to embed socio-cultural and gender based strategies [30]. In terms of non-health system factors, increased community engagement in MNCH program development and delivery, redress of inequalities, and informed health seeking behaviors [30].

#### Study limitations

The findings of the study are limited by the cross sectional and geographical limitations of the research. There was no opportunity to return to the communities to validate the themes, which would strengthen the credibility and trustworthiness aspects of the study. The translation from local languages to English was reliant upon the capacities of the local research team members, so there may be some limitations and incongruencies in this aspect of the work.

#### Implications of findings

The findings of this study reached beyond the statistical and often objective reporting of the mainstream government documents (i.e., cited above). This study put a story to the experience and personal insights into the situation which lacks in the traditional policy documents. The study situates MNCH not in the statistics, but in the lived experiences of many of the women and their communities. It calls for programs which not only change the care environment, but the cultural understandings of MNCH. It highlights the disconnect of programming from the reality of the women especially in areas of gender and culture. It challenges the behaviors of care providers and decision makers on professional and ethical matters. The findings are foundational for future replication research in other areas of Tanzania. In addition, it challenges researchers to undertake studies which consider cultural and gender aspect in MNCH in Tanzania. Another key area for future research is the quality of care and care provider-patient relationship which was a recurring issue in the data.

## Conclusion

This study engaged the community through a series of community dialogues and interviews on outlining a preferred future for MNCH. Through these two approaches, we accessed 132 community participants in the dialogues and ten female clients in the interviews. It was evident from the various sources that health services alone would not determine the successes and achieve the necessary changes for MNCH. Rather the complexities of social, economic, gender, cultural, and political interactions were collectively seen as critical determinants. We also heard that the challenges ranged from simple to complex; from individual to community; as well as from adolescence to end of child-bearing ages. Lack and/or inequitable distribution of resources and minimal control of resources by the target group of women were seen as a significant impediments to progressive and responsive MNCH.

The visions for MNCH were likely the most inspiring and overwhelming contributions to the baseline assessment. Across the spectrum of informants we heard about a future with well-resourced MNCH facilities properly staffed and operated. We heard about the preferred future of ethical, safe, barrier free, and patient-centered MNCH. Over one-quarter of the comments related to issues in MNCH, with nearly another one-quarter relating to experiences (or examples) of the MNCH care continuum. The 45 themes/subthemes were reconsidered and yielded a series of lessons learned. Three main lessons learned were ‘take aways’ from the words of the women we heard.

### What took you so long to come?

Many of the issues and concerns revealed in the baseline assessment are longstanding and have remained unaddressed. As a result we realized that there is no simple fix or answer to issues that have huge social, economic, cultural, and historical roots. Throughout the baseline assessment evidence of poor care quality, underservicing, and questionable capacities was seen. Hence, this lesson is critical as we move forward with regards to the change processes and potential barriers to realizing the MKSHI goals. However, the corollary lesson learned was that there was hope that this time might result in making things better for the women and children. The participants remained optimistic that their right to safe and quality MNCH would come with time and effort. This is an important insight for MNCH programs generally that the solutions must be inter-sectorally based and embedded in an understanding of change.

### How do we know what you know?

The MNCH environment is dynamic and filled with evidence for practice. Patients both indicated a desire to be more knowledgeable and use knowledge more effectively to have better outcomes. Through the qualitative (and quantitative which will be reported in a future article) components of the baseline assessment we gained more evidence of the inequities of knowledge distribution. The lesson learned is that we cannot assume that the knowledge is either ‘out there’ or understood. At times there is a lack of information, whereas at other times there is an overload. Within an ethical MNCH system there was a sense that the best information, the best practices, the best equipment, and the best trained providers would be possible. The sharing of knowledge and the achievement of knowledgeable users of the MNCH systems is a goal that goes far beyond this project and should be high on the agendas of health providers, researchers, and policy makers.

### How will it change for our daughters?

The third lesson learned came from a passionate plea for a better future. The lesson learned was that there is a future vision for MNCH. It was as if the baseline assessment captured this vision, laid the start of the path to the future, and, more importantly, created a sense of responsibility for making the difference for the daughters and granddaughters. The awareness of all participants of the issues, challenges and deficits did not take away from their intention and dream to see positive changes in MNCH. The connection of women through generations and gender creates a solid foundation for MNCH programs.

This final lesson was felt strongly as the most revealing and powerful as it emanated from the women themselves who were envisioning better MNCH programs and services not for themselves but FOR THEIR DAUGHTERS. In this they were heard!

## Abbreviations

IMR: infant mortality rate
MDG: Millennium Development Goal
MKSHI: Mama Kwanza Socio-economic Health Initiative
MMR: maternal mortality rate
MNCH: maternal newborn child health
NMR: neonatal mortality rate
